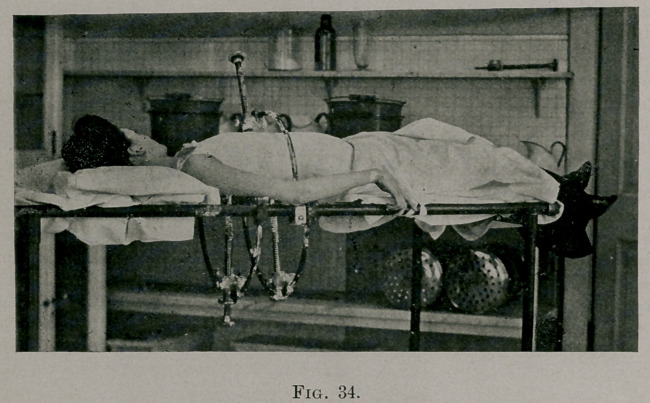# Study of a Case of Lateral Curvature of the Spine. A Report on an Operation for the Deformity

**Published:** 1904-08

**Authors:** Michael Hoke

**Affiliations:** Atlanta, Ga.


ATLANTA

### Journal-Record of Medicine.

**Successor to Atlanta Medical and Surgical Journal, Established 1855, and Southern Medical Record, Established 1870.**

VOL. VI. AUGUST, 1904. No. 5.

BERNARD WOLFF, M.D., EDITOR, Nos. 319-20 Prudential.

Published Monthly.

M. B. HUTCHINS, M.D., BUSINESS MANAGER, 1007-1008 Century Bldg.

### *ORIGINAL COMMUNICATIONS.*

### STUDY OF A CASE OF LATERAL CURVATURE OF THE SPINE: A REPORT ON AN OPERATION FOR THE DEFORMITY.*

FROM THE ORTHOPEDIC DEPARTMENT OF THE PRESBYTERIAN HOSPITAL.

By MICHAEL HOKE, M.D.,

ATLANTA, GA.

(CONCLUDED FROM JULY NUMBER.)

The same story is told by photographs taken at the end of seven months' exercises. (See Fig. 23.) She is standing in the best posture she can assume. The head is held straight. The spine is straight from first cervical to the fourth dorsal, shoulders on a level, the left hip not very prominent, the contour of the left side more natural, the lumbar curve flatter, the spine less curved from the fourth dorsal to sacrum, the trunk vertically above the hips. The

*Presented by invitation at the meeting of the American Orthopedic Association Washington, D. C., May 11-14, 1903.

improvement above the fourth dorsal is permanent, for there was no bone deformity: below, except the increased flexibility, there is no permanent improvement. The improvement is postural entirely, due to a better relation of the elements to one another having been

FIG. 23.

produced by the exercises (stretching contracted ligaments and toning up the muscles). When the picture was taken she was in a state of muscular tension to hold the posture. *Fig. 19 is a photograph taken two minutes after Fig. 23.* See how the body has twisted to the right under the influence of obliquely acting body-weight until it came to a position of rest. The exercises toned up the muscles, increased their power, produced co-ordination, but no human being can or will stand, walk, or sit in a state of muscular strain. A position can not be held which requires a constant muscle contraction for its assumption. The improved posture in this case could be lost in a few minutes, and was, as shown in the photographs. Fig. 23 represents what would be the posture for a few minutes. Fig. 19 would be the posture the remainder of the day, so long as she sat or stood up. *The bone deformity of each element was still present*, the deforming forces still acting as strongly as before. Fig. 3 was taken a few minutes after Fig. 23. The body must twist to the right.

The depression in the back to the left of the chest in front and below the left scapula (normally, a prominence making the shoulder glide forward), and the buckling backward of the ribs to the right of the spine in front of and below the right scapula (normally not so prominent and equal in elevation to the same area on the left, making the right arm glide forward), meant that in this case the left extremity must glide backward, and the right extremity forward, and the weight of these two extremities (a good part of the superincumbent weight) must of necessity act in an oblique manner upon the spine, and, as long as they acted thus, must they keep active a twisting force. This must continue until the shapes, curves of the ribs in question, be restored to something like the normal. Nor could the head be balanced in position until the body beneath should be better balanced. The weight of each thoracic element must, too, be eccentric with reference to the spine (from the shapes of them). Each element would twist upon the spine to the right. Thus the whole thorax would twist.

The question at once came up, why not put the patient in some form of removable jacket, so that exercise could be taken, and preserve the upright position? That would have been done; *but, unless the shoulders were put* in the jacket, the position could not be held. When a jacket was put on up to the armpits, the left shoulder would slide backwards over it, the right shoulder forward, the prominence of the ribs on right side behind appeared increased by the thickness of the plaster. In other words, though not so badly twisted with apparatus as without it, the patient, even with apparatus on, presented a badly *deformed appearance*.

The problem resolved itself into this: Each element, from the fifth inclusive down, instead of being symmetrical, as in Fig. 20, was now deformed into Fig. 21. Then each element from the fifth inclusive down must be changed into Fig. 20, or as near it as possible by some means. This could not be done by plaster; therefore, an operation must be devised.

In planning an operative procedure at this time, two things were dominant: first, to cut the ribs on the left side; second, to cut them without puncturing the pleura. The first could be done, and the second avoided, if the ribs could be successfully shelled out of their periosteal covering at the site at which it was desirable to cut them.

Six dogs were operated upon. The results of these operations demonstrated that in operating upon dogs the following things were true:

First, after the skeleton of the dog's back had been exposed by incising skin and muscles, it was possible to shell a number of ribs out of their periosteum and to divide them each in several places, if so desired.

FIG. 24.

Second, that the periosteum (pleura) on the inner face of the ribs is not so adherent to the ribs as on the outer face (a fortunate circumstance); that the pleural periosteum was attached firmly only to the lips of the borders of the ribs.

Third, there was much danger of puncturing the pleura.

Fourth, the pleura was punctured several times, air was sucked in. The inrushing air was stopped by folding a piece of tissue over the place, and stitching back the periosteum over the rib.

Fifth, where there was much air sucked in through the punctured pleura, the respiration became labored.

Sixth, the hemorrhage from the periosteum and rib amounted to nothing, not more than one or two clamps being required in several rib sections.

Seventh, the best incision through the periosteum is an H-shaped incision—see Fig. 24—rather than only a longitudinal one, for the reason that, when the shaded areas *a* and *b* were reflected back, the remaining periosteal attachment on the outer surface of the rib offered no resistance to stripping the inner face of the rib; whereas in the longitudinal incision troublesome resistance was offered.

Eighth, there is no danger of injuring the intercostal arteries and nerves; they are reflected out of the way as the periosteum is detached.

Ninth, evidently no pain follows the operation other than to be expected from any wound. The dogs breathe quietly and naturally.

One dog died the night of the operation. It was not ascertained why he died. There was no hemorrhage at the operation, nor was his pleura punctured. Unfortunately, his body was immediately thrown out by the janitor before an autopsy could be made.

*Notes of Operation Done on the Cadaver.*

An incision was made through the skin fat and fascia from a point a little to the left of the spine of the fourth dorsal vertebra downwards and outwards to a point a little below and external to the lower angle of the left scapula. A second incision was begun at the lower angle of the scapula, and carried towards the spine of the second lumbar vertebra. A small triangular area just below and internal to the lower angle of the scapula, bordered below by the upper edge of the latissimus dorsi, above by the lower fibres of the trapezius, and externally by the last inch or inch and a half of the posterior border of the scapula, was sought as soon as the skin incisions were made. By going through a thin plane of fascia here, the thoracic wall was exposed to view. It is the only area on the back not covered by muscle. Then by blunt dissection, using the fingers, it was very easy to lift up the trapezius and latissimus dorsi muscles from the thoracic wall, exposing the ribs covered only by loose areolar tissue. By retracting these muscles without cutting them, the fifth, sixth, seventh, eighth, and ninth left ribs were accessible for operation from the angle of the ribs to the attachments of the serratus magnus.

To expose the entire side from the fourth rib down to the twelfth, it was necessary to cut the trapezius across its fibres for about two inches and the latissimus dorsi for about five inches. With this incision the entire left half of the back and the side of the thorax were easily accessible. The hand could be passed up into the axilla, and by retracting the shoulder outwards and forwards the ribs beneath the scapula were accessible for operation.

The rib from its angle to the head could not be operated upon. The tendinous fibres which attach the deep back muscles to the ribs plunge into the rib. These fibres could not be stripped off. There was no detachable periosteal membrane, such as covers the other bony part of the rib. The part of the ribs that could be operated upon extended from the angle to the attachment of the serratus magnus.

The knife was laid aside after the ribs were exposed to view. At a selected site the |—| shaped incision was made with the writer's periosteal elevator No. 1 (see Fig. 25) through the periosteum. With elevator No. 2 (see Fig. 25) the flaps *a* and *b* were rapidly stripped off the outer surface of the rib to its borders.

Though rapid work may be done from this point on, it was necessary to use the most extreme care. With elevators No. 3 (right and left) the periosteum was stripped off the upper and lower borders of the rib to the inner lip. The operator must cling close to the bone with the edge of the instrument, scraping the bone. In this way puncturing the pleura was avoided.

FIG. 25.

Elevators No. 4 (left and right), with longer shanks than No. 3, were used to strip the inner face of the rib of the pleural periosteum. The pleura and periosteum are here the same thin tissue; one can see through it.

The periosteum was attached to the inner lip of the upper and lower borders. Between these lines, or the inner face, it lay in close contact with the rib, but was not attached, as the periosteum is, on the outer surface of the rib and at the borders. It would seem that nature had prepared the way for this operation; for, if the pleural periosteum were as firmly adherent as the outer periosteum, it would be practically impossible to do the operation on account of the time it would take to safely shell out the ribs.

After the section of rib had been shelled out, the operator could do as he wished with the cleaned bone without entering the pleural cavity.

The intercostal blood-vessels and nerves were not injured in the periosteal dissection. The instrument was always between them and the bone of the rib. The periosteum could be stitched over the rib, if so desired.

*Instruments for the Periosteal Dissection.*—A number of instruments were designed and then discarded. Finally, the ones shown in Fig. 25 were designed, and proved to be perfectly satisfactory. Indeed, it would be impossible to do the periosteal dissection with facility without them.

The handle of each, except No. 2, is a little bigger in diameter than a pencil. In using them they are held in the hand just as one holds a pencil in writing. Thus one obtains the advantage of the hand's best tactile judgment in stripping the rib where this procedure is most difficult.

No. 1 has a cutting edge, rounded and sharp. It is used to make the incision through the periosteum. Its advantage over a knife is that it has no point and is much heavier. Its weight necessitates less pressure to be made in making the periosteal incision, thereby decreasing the danger of the instrument slipping and cutting into the lung cavity.

No. 2 through its curved beak enables one to apply the power of stripping effort directly upon the bone. Here, again, the danger of slipping is practically obviated. The beak is one-half inch wide, so that it takes only very few strokes to bare the outer surface of the rib.

No. 3 (right and left) are shaped like golf-sticks. They are used to strip the periosteum from the borders of the ribs. The curve of the shank is such that without changing the posture of the operator's hand the instrument glides over the borders of the rib.

No. 4 (right and left) have longer shanks than No. 3, and are used like No. 3, only, by virtue of their long shanks, one is enabled to strip the inner face of the rib easily.

In using Nos. 3 and 4, they are handled as one uses a pencil in making an up and down stroke, moving the fingers chiefly, the wrist a little. Note the thickness of the shank and its bevelled character. By hugging the bone with the scraping edge, this thick bevelled shank depresses the membrane from the bone, decreasing the danger of cutting through.

No. 5 is a rib-punch with which one can punch a hole in fragments if it be desired to wire them.

No. 6 is an extremely useful costotome. The cutting part of the instrument is at right angles to the handle.

December 3 the patient returned to undergo the first operation. Her condition was good.

Fig. 23 shows the best position she could hold for a minute or two, by much tension. This position she could not hold longer. Fig. 19 shows the body shape as she stood naturally, as she walked. In a jacket the deformity was still just as distressing in appearance. Fig. 3 shows the patient bending forward, the bone deformity of the thorax still present. These photographs were taken within a few minutes of each other. They show that no result but flexibility had been obtained, that the structural deformity of the chest, as above explained in detail, forced the trunk from its weight to twist to the right.

*First Operation, Dec. 6, 1902.*—Usual preparation for operation. Ether. Rubber gloves worn by operator and assistants. Patient lay on her stomach. Beginning at the spine of the fifth vertebra, the incision extended downwards and outwards to a point a little below and external to the inferior angle of the scapula. The second incision extended from the scapula angle towards the twelfth dorsal vertebra. The triangular area was exposed; with the fingers the latissimus dorsi and trapezius were separated. Long clamps were applied to the trapezius perpendicular to the direction of its lower fibres. The trapezius was divided between these clamps for two inches. The rhomboideus major was coextensively cut. By retracting the skin and muscles and holding the upper extremity forwards and outwards the skeleton of the thorax was exposed, the ribs glis-

FIG. Z.

tening beneath a thin layer of areolar tissue. The fifth, sixth, seventh and eighth ribs were selected for operation, because, these being rotated forward, the most of their normal curve having been straightened more than the others, they corresponded to the deepest sunken area in front of and just below the tip of the shoulder-blade; because, as long as that area was sunken in, the plane of the chest-wall would be such that the left shoulder must glide backwards towards the spine; and because the position of these ribs and their attachment to the spine opposed structurally the counter-rotation efforts. If the sunken area were filled in, then the shoulder must glide forwards and its weight would be properly applied to the spine, and that much obliquely applied superincumbent weight overcome: if the area were filled in, each element so operated upon would be nearer the symmetrical shape, and the weight of such element operated upon would fall less obliquely upon the spine. If by the operation the line of action of force $z$ were changed to $z'$ (Fig. 22), then the forces of the elements (ribs) applied to the spine would be more nearly in equilibrium, rib resistance to counter-rotation would be overcome, "rotation flexibility" would be produced. From these things the trunk would be better balanced.

The incision described above was made in the periosteum. The periosteum or the outer surface of the rib for one and a quarter inches was peeled off with elevator No. 2, as near the angle as possible.

Fig. Z shows on *cadaver* the method of stripping rib of its periosteum.

FIG. 26.

Elevator No. 1 was used to strip the inner surface of the rib. (In the dog operations these two instruments were used, and sufficed for the task. The first operation on the patient was done with these two periosteal instruments. One can imagine the discomfort the operator experienced when he found the ribs so crowded together that the blade of No. 1 could hardly be used on the inner face of the rib. It was extremely difficult to strip the ribs' inner surface with a straight instrument without cutting through the pleura with each stroke. But it was finally successfully accomplished.) There was very little bleeding, which stopped as soon as the periosteum was entirely loosened.

After the desired segment of each rib had been shelled out of its periosteum, strips of gauze were passed under the ribs. The ribs were then divided by bone forceps between the gauze strips. A small piece was cut off the fragments distal to the spine, as shown in Fig. 18. An assistant lifted the cut ends by traction on the gauze strips, preventing possible tearing of the pleura by the cut ends moving with the respiratory effort. Notches were cut into the upper and lower borders of each fragment. (See Fig. 26.)

An assistant then made counter-rotation pressure upon the prominence on the right of the spine. The distal fragment was lapped over the fragment nearest the spine. The ends were wired with silver wire catching in the notches. By this rotation and overlapping of the rib fragments the change in each element was as seen in Fig. 27.

Examine the photograph (Fig. 32) to see the actual change.

After the fragments were wired, the periosteum of three segments was stitched back with fine silk. The periosteum of the fourth segments could not be stitched, owing to retraction and contraction of the intercostal muscles.

Two mattress sutures of silk were used to reunite the divided portion of the trapezius. Three vessels were tied in the operation. The skin was sewed up with a subcutaneous stitch. Patient was put in a plaster jacket. It took three hours and thirty minutes to do the operation, from the moment she was placed on the table until she was taken off.

There was a good deal of shock. This shock was produced by several things: prolonged anesthesia, lying on the belly, and the forceful effort to counter-rotate the spine after the ribs had been cut, and the patient's lack of reserve heart power.

As soon as the patient was put to bed, lying on her back, she rallied, and was soon in good condition.

*Notes from Bedside Chart.*

*First Day.*—Badly nauseated, vomiting, pain in abdomen; highest temperature, pulse and respiration, 99½, 136, 50.

*Second Day.*—Badly nauseated, suffered much from abdominal pain; highest temperature, pulse, and respiration, 100, 134, 34.

*Third Day.*—Nauseated, nervous; highest temperature, pulse, and respiration, 100, 118, 26.

*Fourth Day.*—Some nausea and vomiting, better, quiet; highest temperature, pulse, and respiration, 100 2-5 120, 20. Menstruating.

*Fifth Day.*—Pain in stomach; highest temperature, pulse, and respiration, 99 3-4, 108, 20. Nervous.

*Sixth Day.*—Menstruation ceasing, slept well, comfortable; highest temperature, pulse, and respiration, 100, 110, 18.

*Seventh Day.*—Pulse, 87; temperature, 99; respiration, 18. After the seventh there was no discomfort. At no time was there any indication of pleural friction.

*Fourteenth Day.*—Stitches in skin were removed. Healing perfect. In the photograph the scar appears wide, as it stretched. In the fourth week the jacket was changed and the patient was allowed to go home.

*Jan. 29, 1903.*—Returned to-day for jacket. The area operated upon is more filled in, exactly how much it is impossible to know. The left shoulder does not slide backward so easily as it did before the operation. There is not such a great tendency for the body to rotate to right. There is no change in the right side that one can estimate with the eye. The body seems better balanced. The curve of the spine in the mid-dorsal region (as shown by spines of vertebræ) is increased, as would be expected from the mechanics of counter-rotation. Sent home in a jacket.

FIG. 28.

*Feb. 15, 1903.*—Patient returned to-day for second operation. In the first operations difficulties were encountered which would make the repetition of that procedure not advisable. The time consumed was too long, there was too much shock. Could another means be devised to shorten time and facilitate the operation ?

The other periosteal instruments, Nos. 3 and 4 (right and left), were, after having tried and discarded many models, found to make the stripping of the periosteum easy after practice, so that the danger of going through the pleura was minimized and the dependent slowness of the procedure done away with.

Fig. 28 shows a section of a lamb's thorax with the ribs intact; they are stiff and unyielding. Fig. 29 shows the same with ribs operated upon in three places. At each site the rib was not quite severed, all but the inner shell having been removed.

Fig. 30 shows the flexibility of the same side after the operation had been performed.

The degree of flexibility after the operation in animal subjects varied with the toughness of the ribs and the thickness of the shell left. It must vary in the human thorax, and in different parts of the same thorax.

FIG. 29.

The rational thing at this secord operation would have been to operate upon the ribs of the left side, which were not touched in the first operation, left untouched at that operation because the writer did not feel like exposing the patient longer.

But it was feared the patient might not consent to the third operation, which it was seen at this time would be necessary. The hump on the right side of the spine was still present, and projected more with a jacket on than without a jacket. If she should not fail in courage, he hoped to leave her with much of this hump diminished in size. Therefore, he determined to operate upon the right ribs at the second operation.

*Feb. 20, 1903.*—Second operation. Ether. Patient lay on left side. Gloves worn by operator, assistants, and nurses.

It was planned to make two rib sections, as shown in Fig. X from each rib from the fifth to the tenth inclusive, along the lines shown in Fig. 18. The incision made is shown in Fig. 31. The same procedure was done to expose the ribs as was done on the *cadaver* and at the first operation, only the second incision was longer, and a part of the latissimus dorsi was divided across its fibres, as well as a part of the trapezius.

FIG. 30.

Twelve rib sections were made. The average length of time for each section (stripping the periosteum and cutting one section of the rib, leaving the inner table) was three minutes and ten seconds. The periosteum was not stitched back over the gap. The cut in the trapezius was 1½ inches long only. The position while operating under the shoulder-blade was cramped. The latissimus dorsi was cut across its fibres five inches. The

FIG. 31.

incisions in the trapezius and latissimus dorsi were reunited by mattress sutures. Subcutaneous skin suture was used.

Prior to the operation a cast of the back was taken, as the patient bent forward. The hump to the right of the spine was carved from the plaster reproduction of the back made from the cast, and the depression to the left was filled in with plaster. Then a plaster cast of this carved reproduction, of the back only, was made. When the operation was finished, while still under ether, the patient was laid back down in this cast, and strapped to it. A fracture of a number of the ribs was produced. A plaster jacket was applied to the patient lying in this splint, including the splint.

Two hours and three minutes elapsed from the moment the incision was begun until the sewing was finished.

Thirteen minutes were consumed in putting on the jacket. The pleura was not punctured at all. There was no shock, no embarrassed respiration at any time during the operation. The operation was performed in a space of time which could not be regarded as objectionable, and as many ribs were operated upon as will ever need attention in any case. This demonstrated that the procedure could be done successfully without danger to the patient and in a reasonable length of time.

Fig. X shows the mechanics of what was done to each element, and how pressure upon the prominence after the elements had been operated upon would change the shape of each element, the idea being to change the shape by the operation, then to hold the patient in a jacket until new bone should fill in the gap in ribs, thus producing finally a more symmetrical and a solid set of elements. The attempt, one sees, was to flatten the prominence beneath the right shoulder, to change the plane at the thorax at that point, so that the right shoulder would glide backwards instead of forward, and thus make the two shoulder-blades level.

An examination of Fig. 32 shows that the fifth and sixth ribs did not fracture. Notice how prominent they are, and that from that prominence (dotted line at the foot of it) the hump has been symmetrically reduced to the twelfth rib With reference to reducing the hump in the fifth and sixth ribs close to the spine the operation was a failure. But, even with partial change in the fifth and sixth elements, the change produced in the other elements balanced the body better and the right shoulder was easier to hold back, though the tendency for it to glide forward was not entirely overcome. *An effort to counter-rotate was still resisted by the ribs on the left side, untouched at the first operation.* The patient's back was excoriated by the pressure of the plaster. This produced discomfort. Otherwise there was nothing worthy of note in the convalescing period. No such degree of flexibility produced by operating upon the lamb's ribs was obtained here, for the reason that in this patient the outer shell of bone beneath the periosteum was much thicker, and therefore more resistant. Evidently, the operator's desires could not be partly trusted to pressure after the skin incision is sewed. Whatever might be desired must be done completely with the costotome while down upon the ribs. The fractures should have been made complete where they were wanted.

*June 23, 1903.*—Patient returned for the third operation. Her general condition was good. This operation was performed on the 26th of last June. Ether. Gloves. Patient lying on right side. At this operation the technique in getting to the ribs and in shelling them out was the same as in the second operation; hence it is unnecessary to go into details. In this third operation the twelfth rib was fractured during the operation by the fingers after the section of rib had been cut out in one place, the eleventh

FIG. 32.

in two places, the tenth in three places, the ninth in three places, the eighth in three places, the seventh in two places. (The sites operated upon on the seventh and eighth were external to the section made in the first operation.) These fractures were incomplete, "green stick," except at one place on the eighth rib and one place on the tenth. The pleura was punctured once in this operation, the only time in the three operations that this was done. A little air sucked in—very little—the suction of air stopped by folding tissue over the rib and stitching it there. It took one hour and forty minutes to do the operation from the first stroke of the knife to finishing the dressing. The day before the operation the pins of the author's apparatus, used in applying the jackets, were set in just the position desired, so that the proper pressure could be made. As soon as the operation was finished, the patient was transferred to the apparatus. The bandages had been put in water a few minutes before and were ready for use. The application of the jacket took fifteen minutes. There was no shock.

*Note, July* 6.—Ten days since operation. To-day first dressing was made. Union is perfect. Stitches removed. Depression in back much filled out. Patient can move herself somewhat without pain. Condition perfect. Looks better than before she went into the operation. She has had no pain since the operation except a little belly-ache. The highest temperature was 99 4-5; highest pulse, 126. Temperature was normal after the third day. After the dressing was made, the patient was put in the special apparatus and a jacket applied without the use of ether. Ether was not used at any time except when an operation was performed. *It is perfectly easy to rotate the chest in any direction with the hands.* Fig. 34 shows the apparatus for application of the jacket, the patient having been left on the frame long enough to make a photograph. The writer has called this his apparatus for lack of another term of identification. The principles are the same as formerly in use, but the details of construction are different. A jacket can be applied with the patient lying on her face or on a hammock stretched on the gas-pipe frame, the adjustable steel arches can be placed above and below, or the patient can be placed on her back, her hips and shoulders resting on padded boards, as shown in the illustration. The pins, large screws, may be slid along the steel arches to any position desired. The screws, since they revolve in a vertical plane, can

FIG. 33.

be placed so that pressure may be made *upon any part of any element at any angle desired.* In the illustration a padded steel piece an inch wide, and long enough to extend across several ribs, was placed beneath the angles of the right ribs; and the two pins below press upwards at points a half-inch from the ends of the board. In this demonstration each element from the seventh down was subjected to the forces acting as shown in Fig. 33.

*With the flexibility produced by the last operation it was possible to put the*

*patient in this apparatus in such a position that the back was flat* and the front part of the chest perfectly symmetrical.

In a normal thorax the spine is straight, each element is a symmetrical figure, and each element is balanced upon the spine. Thus the whole thorax in a normally developed person is constructed so as to be balanced. Note that the muscles of the trunk are arranged to control the movements of a balanced figure. Were the elements joined behind as they are in front, there would be no flexibility. The ligaments, spinal and costo-spinal, prominently the anterior costo-transverse ligament, the tendinous attachments of the deep muscles from rib to rib, and transverse process to transverse process preserve the poise of the body within the limits of motion possible. The body can not bend further than the ribs permit. Within this limit the ligaments may limit the motion. The muscles execute the motion. The relaxation of a set of muscles can throw the

FIG. 34.

spine out of balance. Thus undue tension is put on the ligaments of the same side. Ligaments stretch from constantly bearing strain. The constant contraction of a muscle or group of muscles and the relaxation of the opposite side can not carry the body in a certain direction *further than the ribs* will permit. If the body bends beyond the natural limit, the ribs bend.

The chain of causes here and in all lateral curvature cases not caused by disease must be this: a group of muscles tires easily, the weight falls obliquely on the spine, rotation and lateral bending of the spine begins, the ligaments stretch, continued obliquely acting weight causes the spine to rotate further, the ribs to go forward and straighten on one side, to rotate backwards and bend backwards on the opposite side, so that finally one has to contend with weak muscles, contracted ligaments, fascia, and tendons on one side, the same stretched on the other side, and a deformed thorax, as above—the expression of the sum of the deformities in the elements composing it. Thus contracted ligaments and soft tissue on one side of the spine form one group of conditions to be treated, the same structures stretched on the other side a second group of conditions to be treated, the weak muscles a third condition, and, last and most difficult, the deformity of each bony element, as described above.

Exercises as a means of developing muscle, to stretch ligaments, to build up the constitution, to produce a poise sense, are most valuable. Nothing else can take their place for these purposes. With jackets one may, if the thorax is put under the proper pressure conditions, obtain correction of bone deformity if the ribs are very flexible and the ligaments not contracted, the correction being trifling or considerable, depending upon how soft the bones may be and how relaxed the ligaments may be.

Hitherto, since no other means was available, exercises and jackets have been applied to cases where they could have no influence upon the bony structure.

In a case with osseous deformity, even after the ligaments, etc., have been stretched and muscles developed, the flattened and anterior displaced ribs are obstructions to rotation *and counter-rotation* of the spine, hence obstructions to straightening the spine as well as being a part of the deformity of the elements.

This operation makes it possible to change bone deformity that can not be affected by plaster. It is thus to be used in the vast majority of cases—the cases that are not simply postural.

The result in this case must be about as clear to those who examine the photographs as to the writer. The case was one of severest curve and osseous deformity. Thus the severest possible test to the surgical procedure has been tested in the first case so treated. The writer is frank to say that the best possible result has not been obtained in this instance. If he could go over this again, he would do to all the ribs in the left side from the fourth inclusive down in one operation as was done to the ribs operated upon in the third operation. He would then, when the patient was in proper condition, operate upon the right side after the same manner done in the second operation, only he would do more and not depend upon the pressure of the jacket (put on immediately after the wound was sewed up) to produce the rib fractures. The above case is the only one ready for this semi-final report.

In Fig. 2 the bad deformity is partly postural and partly structural. In Fig. 23 the postural element (dependent upon contracted ligaments and muscles on the left side, and stretched ligaments and lax muscles on the right side) is gone, but the patient could not hold that improvement. (See Fig. 19, taken two minutes later.) Nor could the writer hold it for her without keeping her in plaster, including the shoulders—a horrible possibility to a woman; and even with such a jacket on, the projection of the hump would have been exaggerated by the thickness of the plaster. The hump and the flattening were still there. (See Fig. 3.)

Fig. 31 shows her upright posture August, 1903, after the three operations. The photographs were taken after she had been standing about ten minutes. The difference had been produced by changing the shapes of the elements, balancing the thorax better by this change. Fig. 32 shows the patient bending forward after the three operations. Contrast Fig. 3 with Fig. 32.

The spine is not straight in the upright posture, but it is straighter and the body outline is far more symmetrical. The posture of the upper extremities is vastly nearer the normal, and to a corresponding extent is the deforming force of their obliquely acting weights incident to their former positions corrected. When the patient lies prone, the spine is straight.

When standing with a light jacket on, very little deformity is perceptible. *The body is very flexible now.* Since the body is so flexible, she will be improved more by jackets put on, with the thorax under the proper pressure conditions.

Since the result is not perfect, she would relapse to a certain extent without a jacket. The writer does not know how far she would relapse without a jacket. It would be interesting, but not fair, to find out. The writer does not believe it is possible for one to make such a deformed thorax perfectly symmetrical again.

The field for the operation's best usefulness must be with the great majority of cases in which a bad figure is dependent upon the deformity of a fewer number of elements than were deformed in this case.

Correction jackets will be worn by the patient, changed once a month, for a while. Then a light removable jacket will be worn, so the patient may exercise (simple extension exercises) daily.

In the examination of lateral curvature cases with osseous deformity one will always find the ribs flat on one side and prominent on the other. The broad mechanical principles of this case are operative in all osseously deformed cases, but there must be an infinitely varied detail, for the curve of the ribs and their angles of inclination to the spine mean, if abnormal, the application of deforming forces to the column and thorax. If one operates, he does so not for disease, but to correct faulty mechanics and deformed anatomy. Thus, given a patient who is in physical condition for the operation, the result must depend upon the operator's interpretation of the forces with which he is contending and the accuracy with which his remedial procedure is executed.

To the writer it seems fair to state that in the future lateral curvature with osseous deformity must be treated as follows:

(1) Exercises must be taken, in order to do away with all contraction of ligaments, fascia, and muscular resistance to flexibility, and to build up the general health. The writer thinks that all the movements of the trunk must be executed in the exercises, regardless of the influence of such exercises upon the rotation of the vertebræ, flexibility being the sole object.

(2) The flattened side of the back must be operated upon so that side forces (ribs) applied to the spine may be so weakened and the ribs made so flexible that the plane of the thorax beneath the shoulder may be changed to as near the normal as possible, the flat ribs recurved towards the normal, and the resistance to rotation reduced or destroyed.

(3) Then a series of jackets must be applied, using the prominent side as a point at which to apply pressure to obtain counter-rotation. These jackets are to be applied until the bone-union in the ribs is perfectly firm.

(4) The curves in the ribs on the prominent side must by operation be restored to as near the normal as possible, in order to do away with the prominence and to restore the natural plane of the thorax beneath the shoulder. The accomplishment of 2 and 3 better balances the thorax.

(5) A series of corrective jackets must be applied until the bone-union in the ribs is firm and all the correction possible obtained.

(6) A removable jacket and daily exercises. How long this jacket should be worn must depend upon the case.

At some time in the future the writer will make another report upon the condition of the patient at the time of such report.

It is not often that one witnesses the cheerful courage this young lady showed in submitting to an untried operation which in this, the first instance, seemed to the writer full of possible difficulties. Hence, in writing the account of this case, it does not seem malapropos to mention one's appreciation of a high courage which expressed itself in a resignation to and confidence in the operator's plans.*

---

*"Operative interference by resection of the ribs has been performed twice by Hoffa on patients, and is said by him to have been suggested by Volkmann in 1899, but not performed by him on the living patient" (*Zeitschr. für orth. Chir.*, 1896, p. 401).

Shaffer advocates a new operation for the cure of rotary lateral curvature which he had performed on the *cadaver.—American Medico-Surgical Bulletin*, Jan. 1, 1894, p. 51, and Feb. 15, 1894, p. 236.

## Figures and Tables

**Fig. 23. f1:**
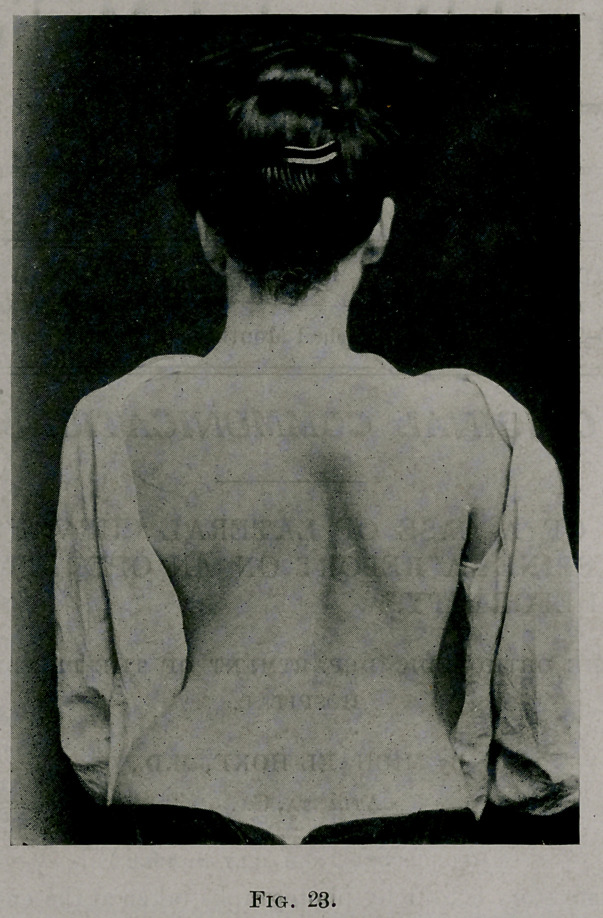


**Fig. 24. f2:**
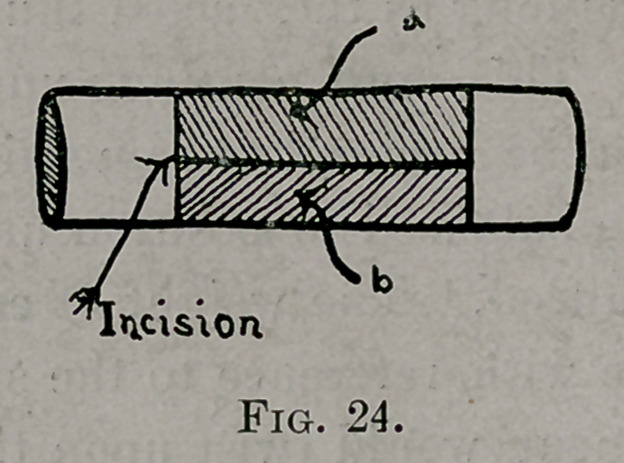


**Fig. 25. f3:**
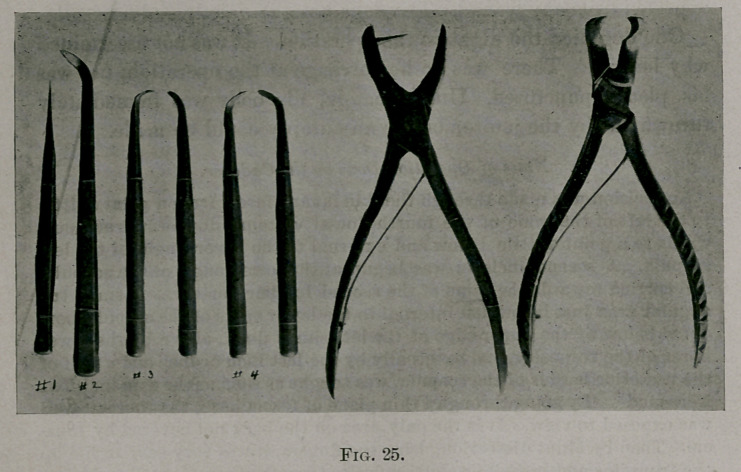


**Fig. Z. f4:**
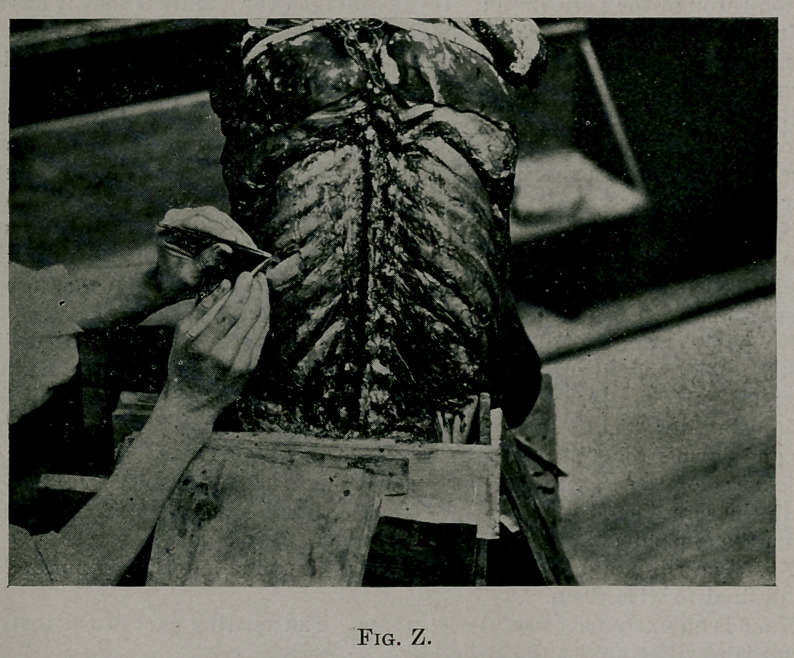


**Fig. 26. f5:**
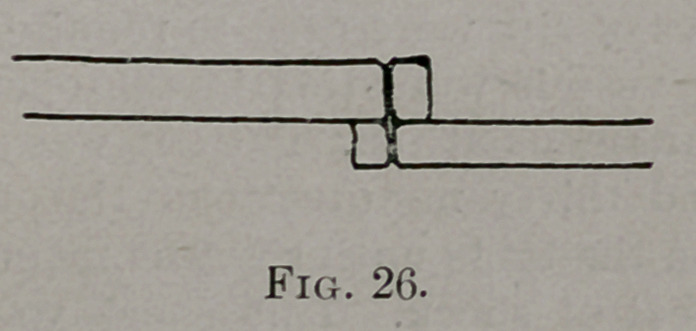


**Fig. 27. f6:**
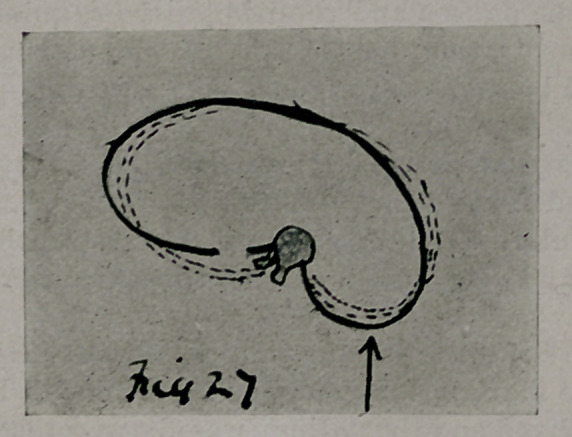


**Fig. 28. f7:**
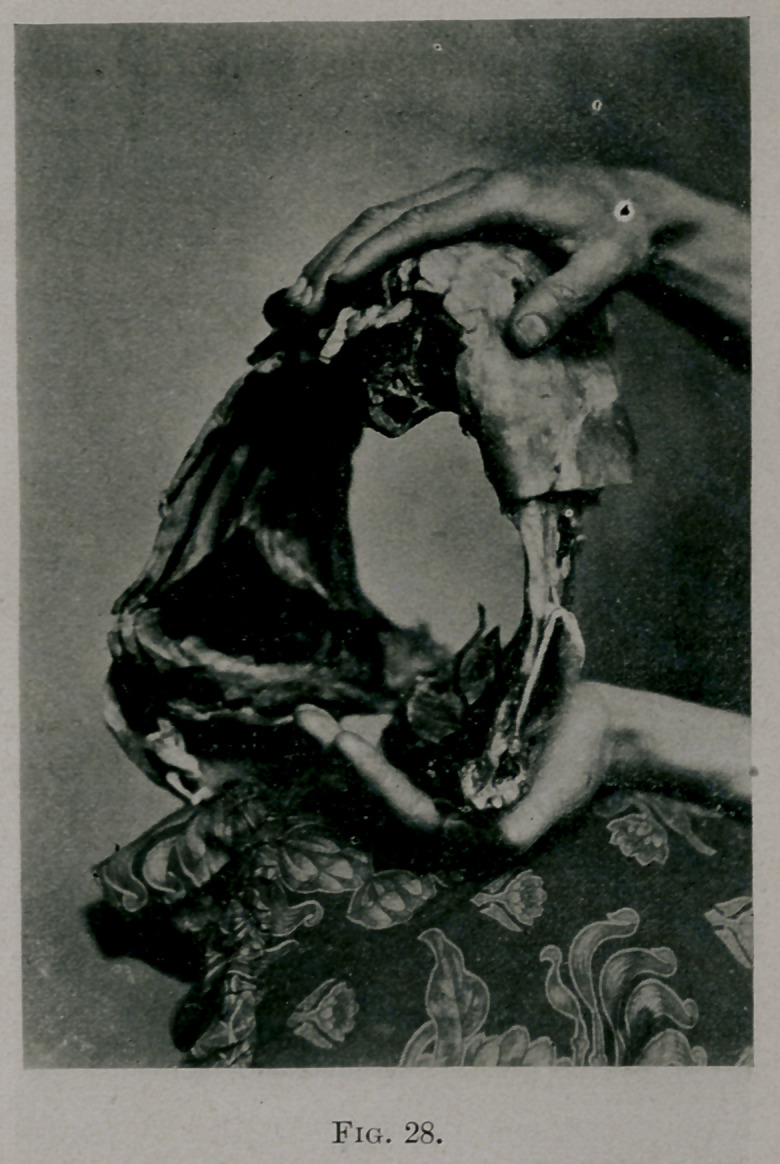


**Fig. 29. f8:**
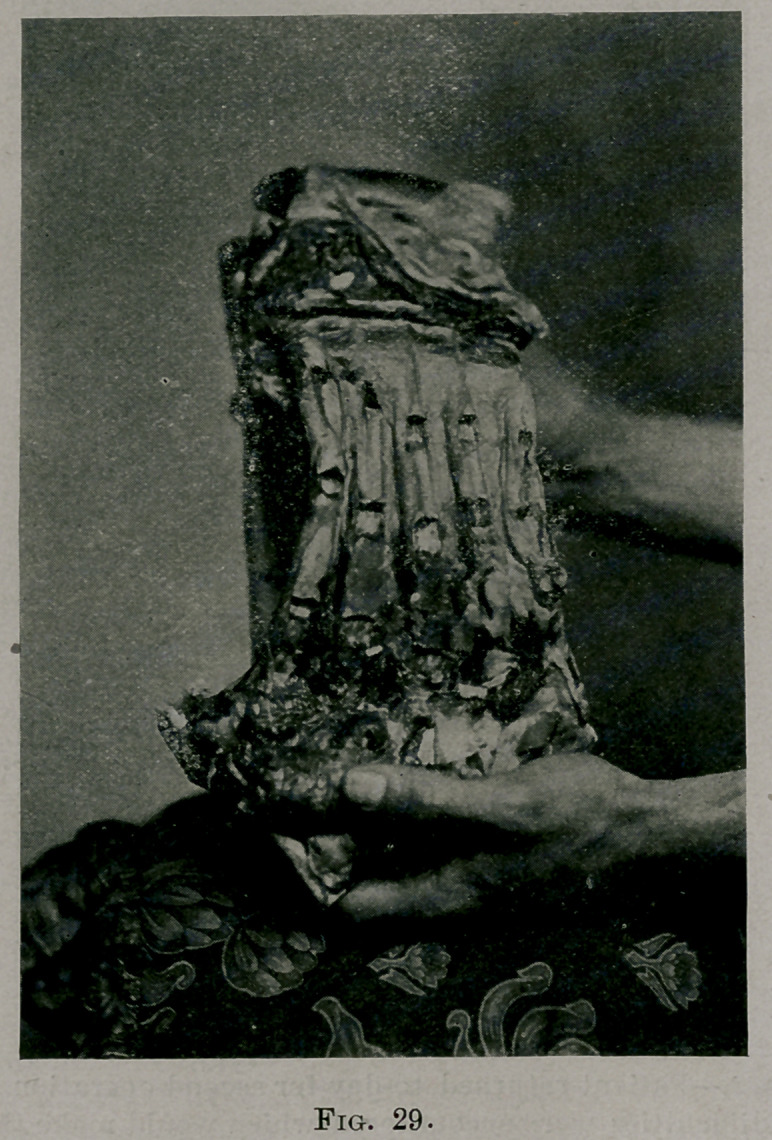


**Fig. 30. f9:**
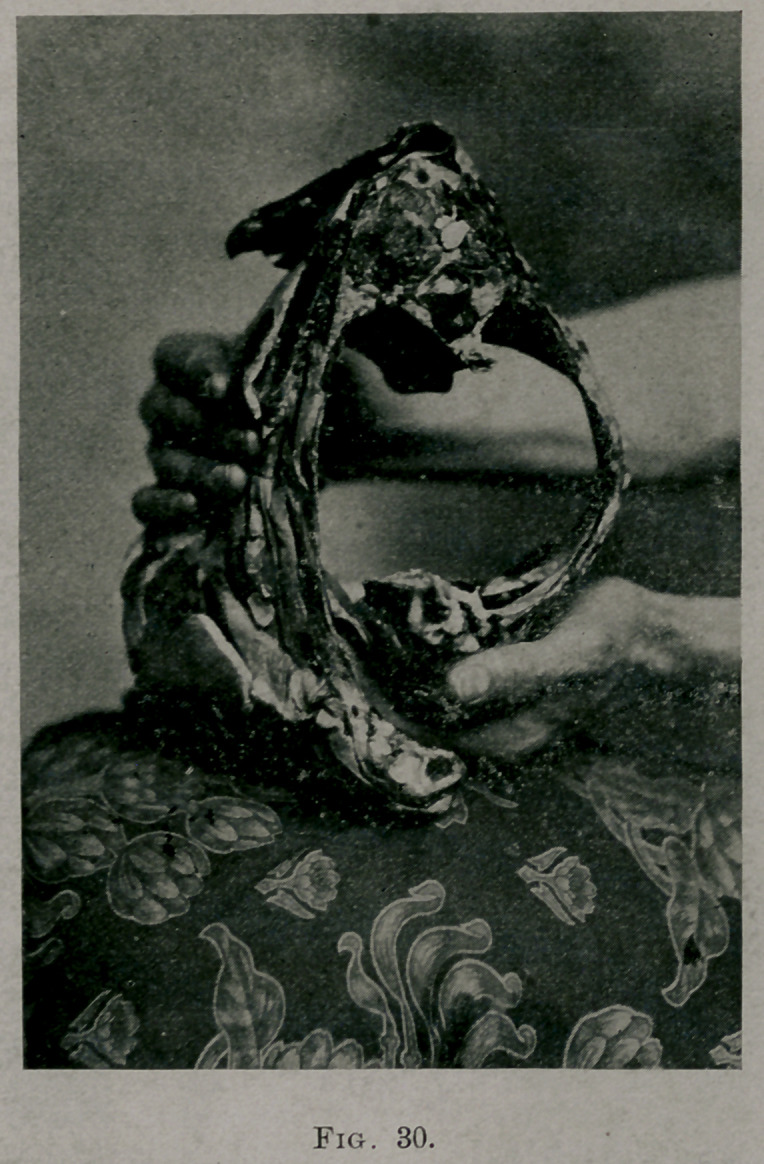


**Fig. 31. f10:**
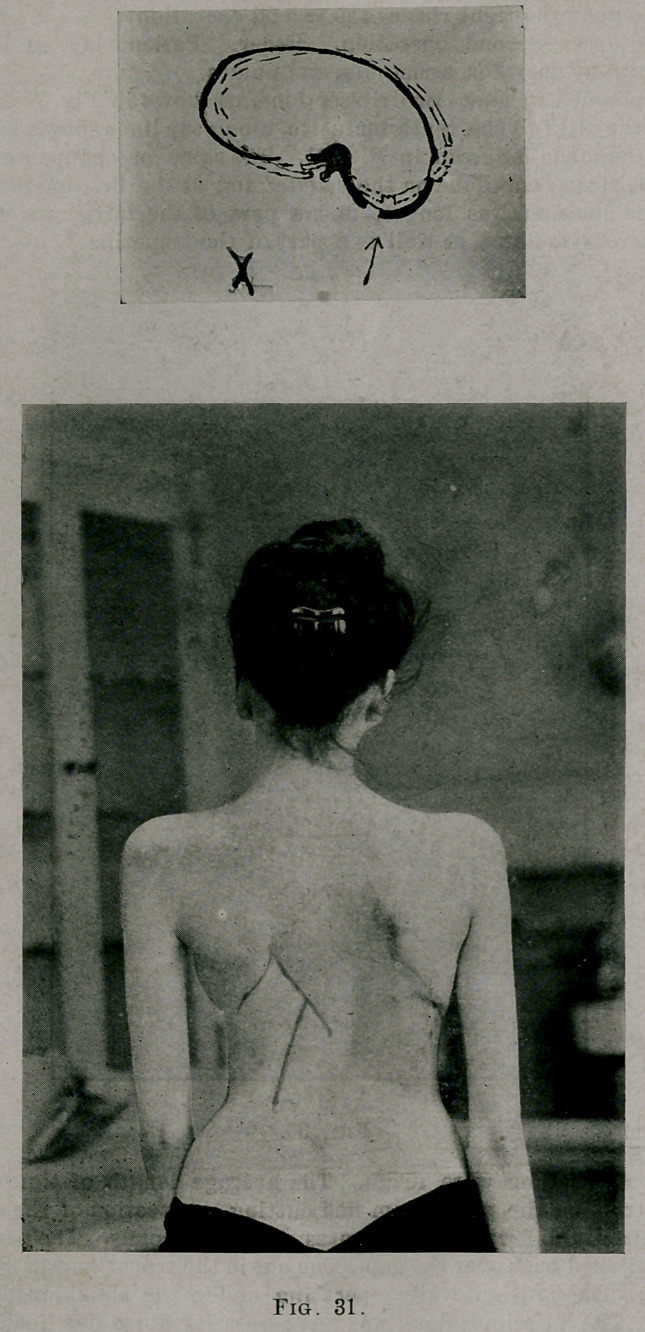


**Fig. 32. f11:**
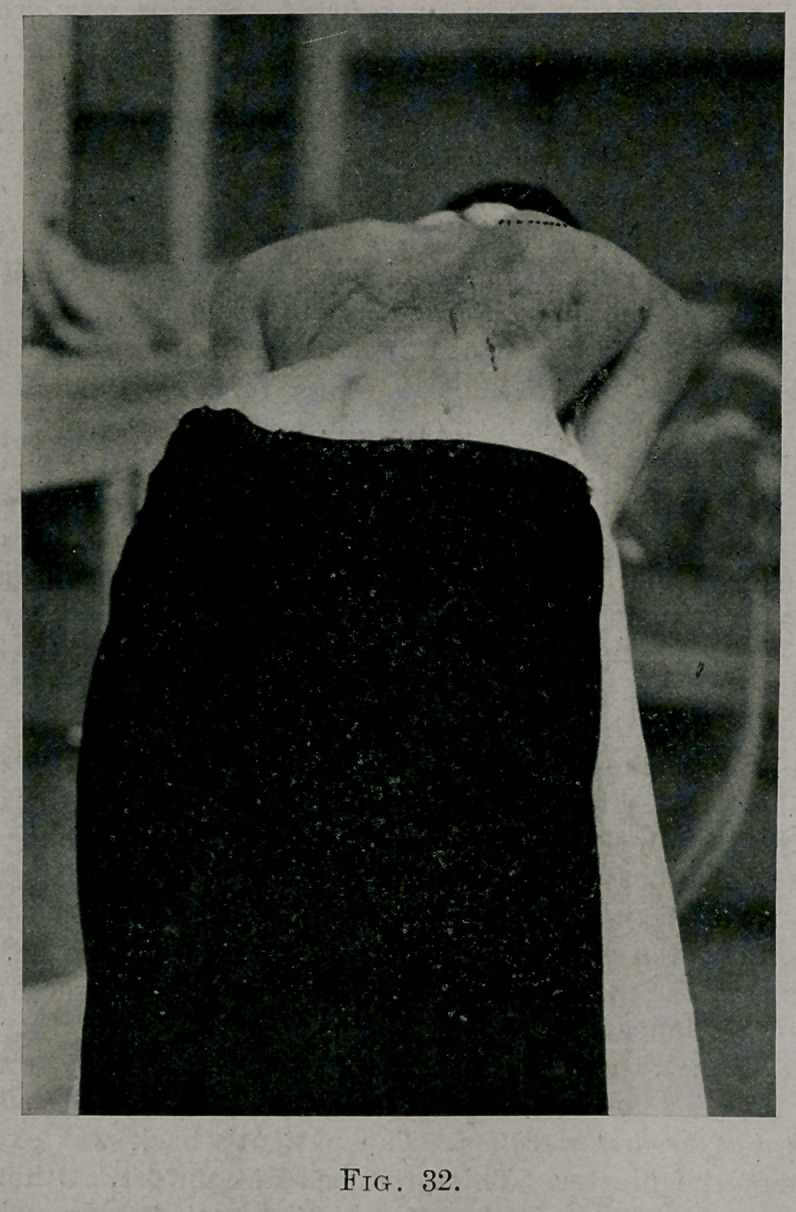


**Fig. 33. f12:**
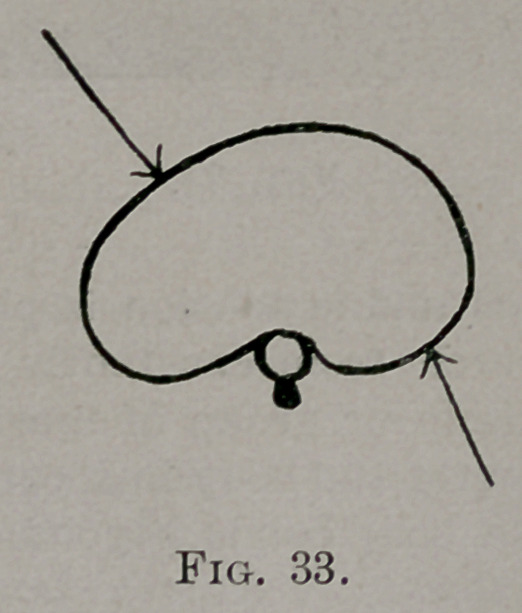


**Fig. 34. f13:**